# Perioperative Complications in Patients with Preeclampsia Undergoing Caesarean Section Surgery

**DOI:** 10.3390/jcm12227050

**Published:** 2023-11-12

**Authors:** Busra Sara Unal, Alicia T. Dennis

**Affiliations:** 1 Hospital Medical Officer, Western Health, St Albans, VIC 3021, Australia; sara.unal@wh.org.au; 2 The Joan Kirner Women’s and Children’s Hospital, St Albans Sunshine Hospital, Western Health, St. Albans, VIC 3021, Australia; 3 Division of Obstetric Anesthesia, Department of Anesthesiology, Perioperative Medicine and Pain Medicine, Brigham and Women’s Hospital, Boston, MA 02115, USA; 4 Department of Critical Care, Obstetrics, Gynaecology and Newborn Health, Melbourne Medical School, The University of Melbourne, Parkville, VIC 3010, Australia; 5 The Royal Women’s Hospital, Parkville, VIC 3052, Australia

**Keywords:** hypertension, heart failure, haemorrhage, perioperative complications, obstetrics, preeclampsia, caesarean section surgery, perioperative medicine, caesarean delivery, anaesthesiology, anaesthesia, pregnancy

## Abstract

Caesarean section has risks of bleeding, infection and thromboembolism, and neuroendocrine-metabolic, and inflammatory-immune responses that may worsen outcomes in patients with preeclampsia. There is little research examining perioperative, as opposed to peripartum, outcomes in patients with preeclampsia. We conducted a single-centrecentre retrospective cohort study of perioperative patients with preeclampsia over an eight-month period to determine the rate of perioperative complication. Seventy-two patients were included. The maternal complication rate was 59.7 per 100 operations (95% CI 48.2 to 70.3%). Severe complications included pulmonary oedema 2 (2.8%), haemorrhage > 1000 mL 5 (6.9%), and blood transfusion 2 (2.8%). Twenty (27.8%) patients had a hospital length of stay ≥7 days. The rate of anaemia (haemoglobin < 110 g/L) on hospital discharge was 42 per 100 operations (95% CI 31.0 to 53.2%). Patient representation rate to hospital after discharge was 23.6% per 100 operations (95% CI 15.3 to 34.6%). There were no maternal deaths. The neonatal complication rate was 38.9 per 100 operations (95% CI 28.9 to 51.1%) with one foetal death. Patients with preeclampsia undergoing caesarean section are a very high-risk surgical group who experience significant perioperative complications. Urgent action is needed to confirm these findings and improve outcomes in these patients.

## 1. Introduction

Preeclampsia is a common hypertensive cardiovascular condition occurring in approximately five to eight percent of all pregnant people worldwide [1]. It is diagnosed clinically when a pregnant person develops new onset hypertension and evidence of end organ dysfunction. It is frequently associated with serious complications such as seizures, stroke, heart failure with acute pulmonary oedema, haemorrhage, liver dysfunction and maternal death [2,3].

Caesarean section surgery is one of the most common surgeries globally [4], and like any major abdominal surgery it comes with the risk of complications including haemorrhage, venous thromboembolism and infection. The caesarean section surgery rate in patients with preeclampsia is nearly twice that of the general obstetric population [5,6]. Patients with preeclampsia who undergo caesarean section surgery are therefore exposed to these additional surgical risks. Furthermore the neuroendocrine-metabolic, and inflammatory-immune response associated with recovery from major surgery may negatively impact recovery in patients with preeclampsia [7]. 

Perioperative medicine is “the multidisciplinary, integrated care of patients from the moment surgery is contemplated through to recovery. It involves preoperative evaluation, risk assessment and preparation, intraoperative care, postoperative care (including monitoring, rehabilitation and post-discharge), communication and handover to primary care or referrer, coordination of personnel and systems and shared decision making [8].” In the area of obstetric surgery, specifically caesarean section surgery, the area of preoperative medicine is still very much in its infancy and patients with comorbidities, such as people with hypertension, are not viewed through the “perioperative medicine” lens. 

Historically, when examining relationships between preeclampsia and pregnancy complications, patients with preeclampsia are rarely divided into those who have major surgery (caesarean section surgery) and those who do not. From an obstetric and midwifery perspective, pregnancy outcomes for patients with preeclampsia have included the mode of birth outcome of caesarean section rather than comparing specifically the outcomes in patients with preeclampsia who also undergo major surgery (caesarean section surgery) with those who do not (vaginal birth). Furthermore, adverse events after caesarean section have been examined generally but rarely stratified according to the comorbidity of preeclampsia [9]. From an anaesthesiology perspective, obstetric patients have, until recently, been a forgotten perioperative population left behind in the emerging subspecialty of perioperative medicine [10]. This is despite these surgical patients presenting with the significant comorbidity of hypertension with organ dysfunction which may also be uncontrolled and complicated by extreme features of organ damage. 

Despite the common co-occurrence of patients with preeclampsia undergoing caesarean section surgery, data specifically focussing on adverse perioperative outcomes for these patients are lacking. This suggests that this key intersection of obstetrics, surgery, anaesthesiology, and perioperative medicine may have been neglected. It is likely that complications in the perioperative period in this group of patients are significant [11,12]. As a first step in addressing this issue the aim of this study was to determine the rate of perioperative complications of patients with preeclampsia, and their babies, who underwent caesarean section surgery. 

## 2. Materials and Methods

### 2.1. Study Setting and Participants 

After Human Research and Ethics Committee approval from the Royal Women’s Hospital, Melbourne, Australia (HREC EC00259 AQA19/34) we conducted a single-centre observational cohort study. The study was conducted in a tertiary referral teaching hospital with over 7000 births per year in a high-income country. Consent was waived due to the retrospective nature of the study without contemporaneous participant recruitment. The study adhered to STROBE guidelines for reporting observational studies [13]. 

We included all patients who were identified by International Coding of Diseases version 10 Australian Modification (ICD10-AM) coding system as having preeclampsia and also had a caesarean section at The Royal Women’s Hospital, Melbourne, Australia from 1 May to 31 December 2019. Patients with non-singleton pregnancies, a history of stimulant use during pregnancy, comorbid conditions such as pre-existing diabetes (excluding gestational diabetes) and pre-existing hypertension, were excluded from the study. 

### 2.2. Defintion of Preeclampsia

Preeclampsia was defined as new-onset hypertension (blood pressure ≥ 140 millimetres of mercury [mmHg] and/or diastolic pressure ≥ 90 mmHg) at 20 weeks’ gestation or greater along with evidence of end organ damage. Evidence of end organ damage included: proteinuria, supported by a urine protein-creatinine ratio ≥ 30 milligram/millimoles; evidence of acute kidney injury as demonstrated by a rise in serum creatinine; liver dysfunction indicated by derangement of liver function tests; the presence of neurological symptoms such as visual disturbance, headache or seizure on history; the presence of haemolysis or thrombocytopaenia on blood film or full blood examination and a diagnosis of fetal growth restriction as demonstrated by serial growth scanning antenatally and/or a diagnosis confirmed by a paediatrician’s assessment after birth [2,3].

Severe features of preeclampsia were defined as: systolic blood pressure ≥ 160 mm Hg and/or diastolic blood pressure ≥ 110 mm Hg on two occasions at least four hours apart (unless antihypertensive therapy is initiated before this time); thrombocytopaenia (platelet count ≤ 100,000 × 10^9^ per litre) on full blood examination; impaired liver function indicated by abnormally elevated blood concentrations of liver enzymes (to twice the upper limit of normal concentration); severe persistent right upper quadrant or epigastric pain unresponsive to medication and not accounted for by alternative diagnoses; renal insufficiency (serum creatinine ≥ 1.1 mg/dL or a doubling of the serum creatinine in the absence of other renal disease); the presence of pulmonary oedema; new-onset headache unresponsive to medication and not accounted for by alternative diagnoses; visual disturbance; eclampsia; the use of magnesium sulphate; a diagnosis of fetal growth restriction (e.g., on serial growth scanning or as confirmed by paediatrician’s assessment) [2,3]. 

### 2.3. Data Collection

Data were extracted from the patient medical records including the Victorian Maternal Health Record, discharge summary, progress notes, anaesthetic record, operative report and drug chart(s). 

### 2.4. Statistical Anaylsis

Data were analysed using SPSS Statistics Version 27 (IBM© SPSS© Statistics Version 24 IBM Corporation 2020, Chicago, IL, USA). Data are presented as mean, standard deviation (SD), median (quartiles of lowest and highest value), or number and percentage as appropriate. The Kolmogorov–Smirnov normality test was performed on continuous variables to determine distribution. Comparisons between variables were performed using unpaired t tests, one-way ANOVA with Tukey’s multiple comparison test, Kruskal–Wallis test with Dunn’s multiple comparisons test, or Mann–Whitney test as appropriate. Proportions were compared using Fisher’s exact test. 95% confidence intervals (CI) are presented as appropriate. 

## 3. Results

### 3.1. Participant Characteristics

**Figure 1:**
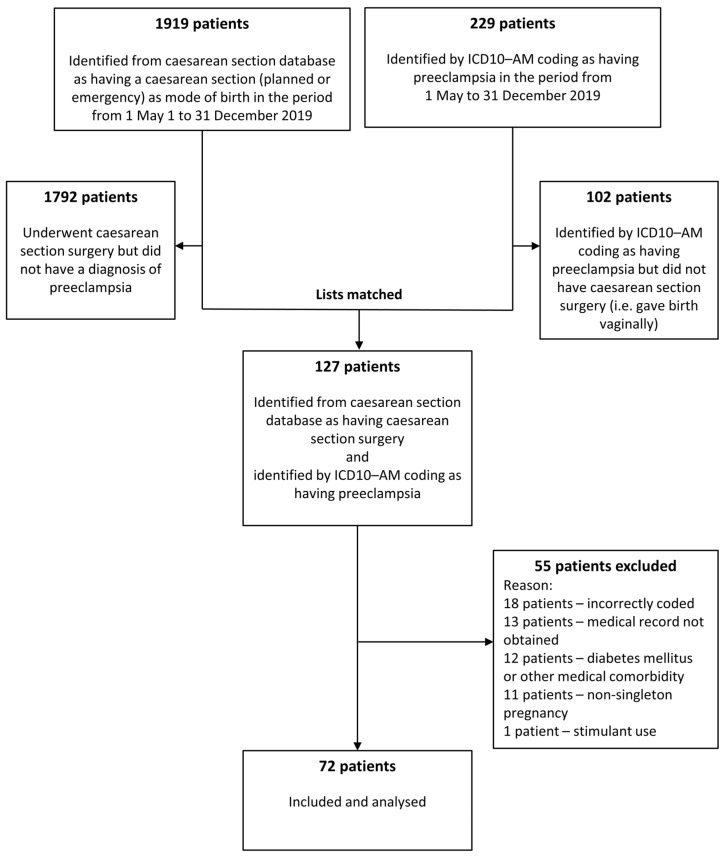
Patient flowchart. ICD10-AM = International Coding of Diseases version 10 Australian Modification.

During the eight-month period 1919 patients had caesarean section surgery and 229 patients had preeclampsia. One hundred and two patients with preeclampsia gave birth vaginally. One hundred and twenty seven patients were identified with preeclampsia and underwent caesarean section surgery (caesarean section rate 55.5%). Fifty-five patients were excluded, resulting in 72 women being included in this dataset (Figure 1). 

Participant characteristics are shown in Table 1. Patients with preeclampsia with severe features underwent caesarean section at an earlier gestation than those without, and two patients demonstrated evidence of uncontrolled diastolic blood pressure. 

Other features of the cohort were that nearly three in four patients had severe preeclampsia, and that preeclampsia was diagnosed antenatally in 50 (69.4%) patients, intraoperatively in 10 (13.9%) patients and postoperatively in 12 (16.7%) patients. 

For patients who were diagnosed in the antenatal period, the median (IQR) gestation at diagnosis was 39 (38, 40) weeks for patients with preeclampsia without severe features, and 37 (33, 38) weeks for patients with severe features (*p* = 0.0014 95% CI 37.1 to 40.4 weeks). Patients with preeclampsia with severe features underwent caesarean section on average between 0.6 and 3.1 weeks earlier than patients without severe failure. 

**Figure 2:**
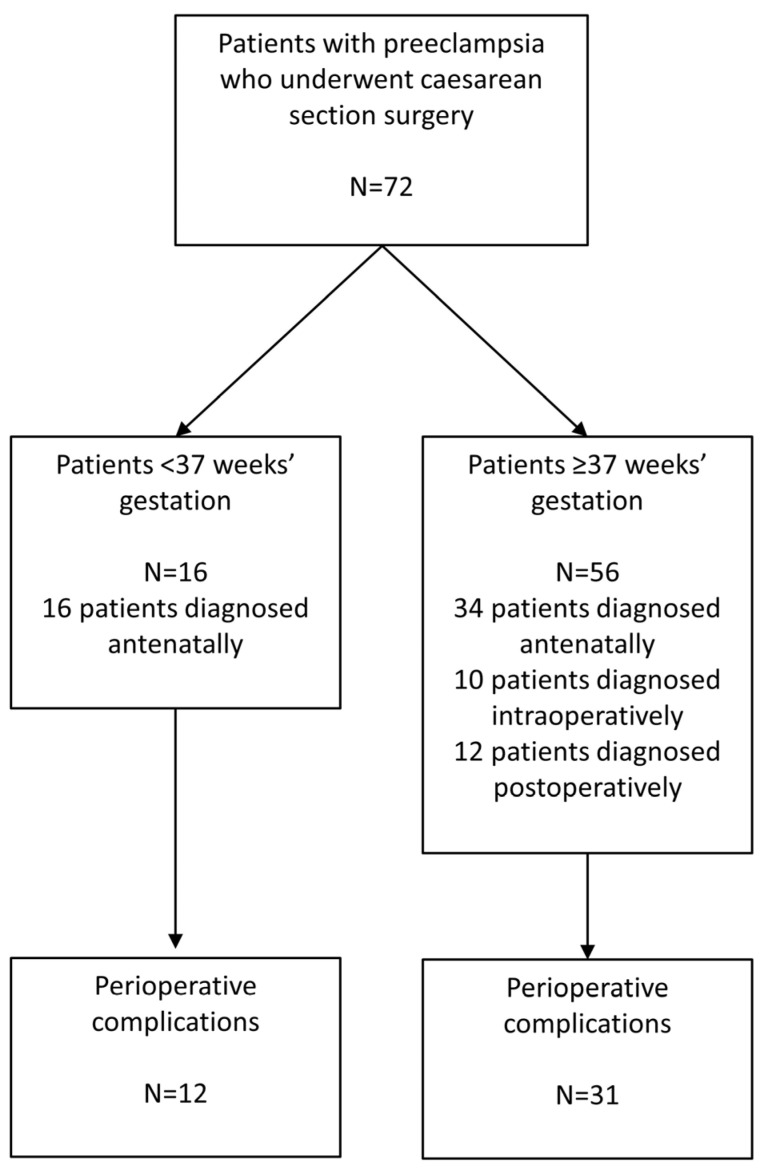
Complication number experienced by patients who underwent caesarean section in the preterm period less than (<) 37 weeks’ gestation, and those who underwent caesarean section at or greater than term (≥) 37 weeks’ gestation.

Figure 2 shows complications for patients who underwent caesarean section in the preterm period and those who underwent caesarean section at term. All patients who underwent surgery when preterm were diagnosed with preeclampsia in the antenatal period compared with patients who underwent surgery at term who were diagnosed antenatally, intraoperatively and postoperatively. Complication rates in patients < 37 weeks’ gestation were similar to those ≥37 weeks’ gestation (75% vs. 55% *p* = 0.2378). There were 10 patients with preeclampsia ≤ 34 weeks’ gestation. Complication rates in these patients were also similar to patients ≥ 37 weeks’ gestation (80% vs. 55% *p* = 0.1797).

Thirty-three (45.8%) patients underwent emergency caesarean section during labour. Forty-five (62.5%) patients were treated with single or combination antihypertensive medication of labetalol, nifedipine and methyldopa prior to surgery and most patients presented for surgery with controlled blood pressure. Three (4.2%) patients were also treated with intravenous magnesium sulphate. Thirty-seven patients (51.4%) continued to be prescribed antihypertensive medication postoperatively, with fifteen patients (20.8%) commencing an additional class of antihypertensive medication after surgery. Seventeen (63.0%) of the 27 patients who did not receive antihypertensive medications prior to surgery commenced antihypertensive medications in the postoperative period. Forty-six women (63.9%) were discharged home on antihypertensive medication. 

### 3.2. Maternal Preoperative, Intraoperative, and Postoperative Complications 

**Figure 3:**
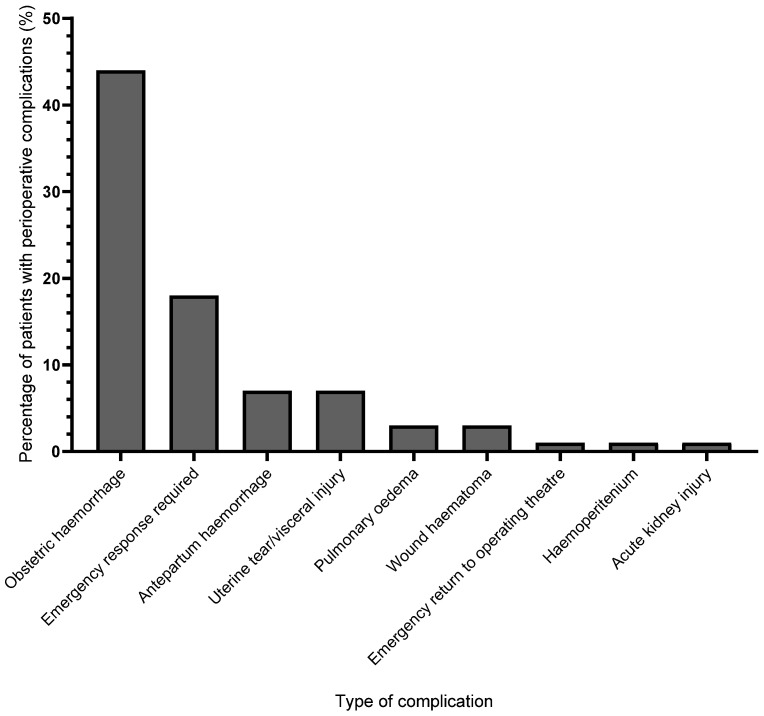
Causes of in-hospital perioperative complications in patients with preeclampsia who underwent caesarean section surgery. Some patients experienced more than one complication. Emergency responses were for hypertension, hypotension, tachypnoea, severe pain or loss of consciousness.

Maternal perioperative complications are shown in Table 2 and Figure 3. The rate of complications for the group was 59.7% per 100 operations (95% CI 48.2 to 70.3%) with 43 of the 72 patients experiencing complications. Overall, we found no difference between the number of complications occurring in patients without severe features of preeclampsia and those with severe failure. Some patients experienced more than one complication. Seven of the 10 patients who were diagnosed with intraoperative preeclampsia also experienced intraoperative haemorrhage. No patients were admitted to the intensive care unit. Six (8.3%) patients with severe features were admitted to a higher acuity unit (high dependency unit). There were no maternal deaths. 

Haemoglobin concentration was measured in 58 (80.6%) patients prior to discharge. The mean (SD) haemoglobin level was 110 (15.6) g/L with 30 (41.2%) patients meeting the definition of anaemia (haemoglobin < 110 g/L). The mean (SD) length of hospital stay was six (2.5) days with no evidence of a difference in length of stay between those people without (5 (1.9) days) and with severe features (6 (2.7) days) (mean difference 0.5 days, 95% CI −1.7 to 0.7 days *p* = 0.4017). Twenty (27.8%) patients had a hospital length of stay of 7 or more days.

The anaesthesia and surgical providers for all patients were either specialist or physician non-specialists (specialists in training). The anaesthesia techniques used were spinal anaesthesia in 34 (47.2%) patients, epidural anaesthesia (epidural top-up) in 33 (45.8%) patients, general anaesthesia in three (94.2%) patients or combined spinal-epidural anaesthesia in two (2.8%) patients. There were no anaesthesia complications reported. 

The complications experienced by patients after discharge are shown in Table 3. Four of the 15 people who presented to the emergency department were readmitted to hospital. The representation rate to hospital, either due to presenting to the emergency department or being readmitted to hospital, within 28 days of discharge, was 23.6 per 100 operations (95% CI 15.3 to 34.6%). 

### 3.3. Neonatal Complications 

Apgar scores in the 71 babies born alive at 1 and 5 min (median (IQR [range]) were 8 (7–9 [3, 9] ) and 9 (8–9 [2, 10]), respectively. The average (SD) birthweight was 2946 (921) g. Forty-five (62.5%) babies were male and 27 (37.5%) were female. 

Neonatal resuscitation was required in 23 (31.9%) with neonatal admission to neonatal intensive care unit or special care nursery required in 28 (38.9%) of the 71 babies (95% CI 28.9 to 51.1%) for complications including non-invasive oxygen therapy (10 babies), invasive ventilation (6 babies), hypoglycaemia management (5 babies), hypothermia management (4) and observations in the setting of prematurity (3). There was no evidence of a difference in the rate of neonatal complications between the group of patients with no severe features and those with severe features (22 out of 55 (40%) vs. 6 out of 17 (35.3%) *p* = 0.7833). There was one fetal death in a patient with preeclampsia with severe features. 

## 4. Discussion

The key findings of this study were that the rate of perioperative maternal complications associated with caesarean section surgery for patients with preeclampsia was 59.7 per 100 operations (95% CI 48.2 to 70.3%) and neonatal complications 38.9 per 100 operations (95% CI 28.9 to 51.1%). The rate of maternal anaemia on discharge from hospital was 42 per 100 operations (95% CI 31.0 to 53.2%). The rate of maternal representation to hospital after discharge was 23.6 per 100 operations (95% CI 15.3 to 34.6%). 

Patients with preeclampsia, regardless of severity, were very high-risk patients undergoing surgery. In the immediate preoperative period nearly 10% of patients had life-threatening complications. Five patients had an antepartum haemorrhage, including two patients with placental abruptions, one of which resulted in a fetal death. One patient was in heart failure with pulmonary oedema. Most patients were undergoing surgery when they had hypertension with severe features suggestive of end organ damage. The use of perioperative magnesium sulphate was very low. 

Patients in our study experienced significant intra- and postoperative complications. Over 40% of patients had significant intraoperative complications, predominantly haemorrhage, and 25% had postoperative complications including sustained hypertension, loss of consciousness and pulmonary oedema. Emergency response calls were required on 21 occasions. Hospital length of stay was nearly one week and when patients were discharged over 40% of them had anaemia. 

Postoperative complications extended beyond patient discharge from hospital, with over one in five patients representing to the emergency department for care. Patients required emergency management for the treatment of hypertension including four patients who needed readmission to hospital to control hypertension. 

The babies born to these patients also suffered significant and life-threatening complications. Nearly one in three babies required resuscitation at birth and sadly there was one fetal death in the group. Over one in three babies were admitted to higher acuity care units, necessitating being separated from their mother.

Moroz and colleagues reported in a large multicentre study of 1,339,397 unstratified patients from the United States a complication rate after caesarean section of 6.7% [9]. Higher rates were reported in teaching hospitals (8.1%) compared with non-teaching hospitals (5.4%) presumably due to more complex patients attending teaching hospitals. The most common complications were haemorrhage (2.0%) and wound complications (0.9%). A 1.7% transfusion rate was reported. Hospital length of stay ≥ seven days occurred in 0.82% of patients. Patients described as having severe preeclampsia, the older term for preeclampsia with severe features, had a relative risk of complications of 2.54 (95% CI 2.29 to 2.71) however the proportion of patients with severe preeclampsia who experienced complications, or a characterization of the complications they experienced, is not presented. 

Bishop and colleagues, in the large African Surgical Outcomes study of 3685 unstratified patients undergoing caesarean section, reported a postoperative complication rate of 17.4% [12]. The most common severe complications were intraoperative and postoperative bleeding reported in 3.5%. Pulmonary oedema occurred in 12 patients (0.33%). Cardiovascular complications were associated with the highest mortality rates with nine patient deaths (43%) out of 21 patients with these complications. This study did not examine complication rates for patients with preeclampsia. 

The rates of complications in our study in patients with preeclampsia are much higher than rates of complications reported in both high income and low and middle income countries. In comparison with Moroz and Bishops’ studies we have reported significantly higher rates of complications: a 59.7% overall rate of complications, a 27.8% rate of hospital length of stay ≥ 7 days, a 6.9% rate of haemorrhage > 1000 mL, a 2.8% transfusion rate, and a 2.8% pulmonary oedema rate. These rates are alarmingly high and suggest that patients with preeclampsia represent an unrecognized high risk surgical group. 

The dual problem of obstetric haemorrhage and preeclampsia, identified in our study, represents a particularly challenging perioperative problem. This is for two reasons. The first is that the usual agents to control haemorrhage such as ergometrine are contraindicated in this group thereby somewhat limiting the pharmacological agents available to manage bleeding. The second is that excessive fluid resuscitation may result in pulmonary oedema [14] due to preserved ejection fraction heart failure in patients with preeclampsia, yet under resuscitation may lead to acute kidney injury [15] and shock. Awareness by the multidisciplinary team that haemorrhage is a significant risk in patients with preeclampsia, early recognition, and management, including blood transfusion if appropriate, are essential. Under the circumstances of perioperative haemorrhage our findings of an anaemia rate of 42% on hospital discharge is significant. It suggests more active treatment of anaemia, such as with iron infusions, is required prior to patient discharge [16].

Finally, our data showing that nearly one in four patients represented to hospital after discharge for the management of complications, suggests that the acute problems these patients experience extend beyond hospital and impact upon their short term recovery. 

## 5. Limitations 

Our study has some limitations. The first is that it is a relatively small single-centre retrospective study. Although the centre is one of the largest obstetric hospitals in Australia and is a tertiary referral and teaching hospital, these data cannot necessarily be extrapolated to other centres.

Secondly, the retrospective nature of the study means that it is fallible to data entry and coding errors, and discrepancies in diagnosis between clinicians, and omission of information from the medical record. These problems may impact upon the recorded outcomes.

Thirdly we did not include patients with multiple pregnancies as we thought this would lead to a confounding of the results as these patients are high risk patients due to these other coexisting conditions. This exclusion may have led us to miss important complications in these patients with preeclampsia. 

Fourthly, we did not include a control group of patients without preeclampsia who underwent caesarean section, although we would expect their complication rate to be low, so we were unable to determine our baseline rate of complications in healthy pregnant patients for comparison. We also did not compare perinatal complications in the total group of patients with preeclampsia stratified for the type of delivery. Future studies should compare these groups specifically.

Finally, we have included all complications which the patients with preeclampsia suffered from. Some of these complications are severe whilst others may be considered less significant. The inclusion of all complications has increased the overall rate of complications. Our study was however one of the first studies to examine perioperative complications specifically in patients with preeclampsia in the perioperative period. 

## 6. Conclusions

Patients with preeclampsia, regardless of its severity, undergoing caesarean section surgery are very high-risk surgical patients. Our findings of very high perioperative maternal complication rates, as well as very high rates of significant obstetric haemorrhage, pulmonary oedema, blood transfusion and hospitalisation equal to or longer than seven days are surprising and concerning. There is an urgent need to confirm these data with larger prospective studies, to understand better why magnesium sulphate is not being used more widely, and if verified, to commence widespread healthcare educational programs and interventions to reduce the morbidity in patients with preeclampsia undergoing caesarean section surgery.

## Figures and Tables

**Table 1:** Participant characteristics.

	No Severe Features *n* = 17	Severe Features *n* = 55	*p* Value (95% CI)
Age (years) ^@^, mean (SD)	32 (5.4)	34 (5.8)	0.2333 **
BMI (kg/m^2^) *^@^, mean (SD)	33 (6.9)	31 (7.2)	0.5582 **
Nulliparous, *n* (%)	15 (20.8%)	40 (55.6%)	0.3267 ^@@^
Previous CS, *n* (%)	2 (2.8%)	13 (18.1%)	0.4953 ^@@^
Gestation at CS (weeks) ^@^, mean (SD)	39 (1.7)	37 (3.4)	0.0036 ** (0.6 to 3.1) ^
Emergency CS, *n* (%)	13 (21.3%)	48 (78.7%)	0.2754 ^@@^
Preoperative haemoglobin (g/L) ^#@^, mean (SD)	127 (14.3)	124 (13.9)	0.4388 **
Preoperative heart rate (1/min), mean (SD)	86 (12.1)	95 (13.4)	0.0364 ** (0.6 to 17.5) ^
Preoperative systolic blood pressure (mmHg), mean (SD)	134 (18.0)	135 (14.2)	0.8303 **
Preoperative diastolic blood pressure (mmHg), mean (SD)	84 (11.8)	82 (11.8)	0.6063 **
Preoperative oxygen saturation (%), median [IQR]	100 [98, 100]	100 [98.5, 100]	0.8967 ^&&^
Preoperative systolic blood pressure >160 mmHg, *n* (%)	0	2 (3.6%)	N/A
Preoperative diastolic blood pressure >110 mmHg, *n* (%)	0		N/A

* Preoperative weight, and body mass index (BMI) at time of surgery was recorded on day of surgery in 60 patients. CS = caesarean section; ^#^ Haemoglobin was measured in 57 patients preoperatively; ^@^ Data are mean (SD), or median [IQR]. ^ 95% CI of mean difference. Vital sign data—whole group *n* = 55, severe features *n* = 41, no severe features *n* = 15. Comparisons (*p* values) are between no severe features group and severe features group. ** Age, BMI, gestation, haemoglobin, heart rate, systolic and diastolic blood pressure comparisons between groups used two-sided unpaired *t* test with Welsh’s correction. ^@@^ Proportion comparisons between groups (nulliparous, previous CS, and emergency CS) used Fisher’s exact test. ^&&^ Oxygen saturation between groups was compared using Mann–Whitney test computing an exact *p* value taking into account ties among variables. N/A = not applicable.

**Table 2:** Maternal preoperative, intraoperative and postoperative complications.

	No Severe Features *n* = 17	Severe Features *n* = 55	*p* Value
**Preoperative complications, *n* (%)**	0	6 (8.3%)	N/A
Antepartum haemorrhage, *n* (%) **	0	5 (6.9%)	
Pulmonary oedema, *n* (%)	0	1 (1.4%)	
**Intraoperative complications, *n* (%)**	9 (12.5%)	23 (31.9%)	0.5775 ^@@^
Obstetric haemorrhage, *n* (%) ^	9 (12.5%)	23 (31.9%)	
Blood loss (mL), mean (SD)	562 (291)	470 (264)	
Blood loss > 1000 mL, *n* (%)	2 (2.8%)	3 (4.2%)	
Blood transfusion, *n* (%)	0	2 (2.7%)	
Uterine tear during surgery, *n* (%)	1 (1.4%)	3 (4.2%)	
Other viscera injury, *n* (%)	0	1 (1.4%)	
**Postoperative complications, *n* (%)**	3 (4.2%)	15 (20.8%)	0.7438 ^@@^
MET ^&^ response required, *n* (%)	2 (2.8%)	11 (15.3%)	
Hypertension, *n* (%)	0	9 (12.5%)	
Hypotension, *n* (%)	0	1 (1.4%)	
Tachypnoea, *n* (%)	0	1 (1.4%)	
Severe pain, *n* (%)	1 (1.4%)	0	
Loss of consciousness, *n* (%)	1 (1.4%)	0	
Wound haematoma, *n* (%)	1 (1.4%)	1 (1.4%)	
Return to operating theatre, *n* (%)	0	1 (1.4%)	
Acute kidney injury, *n* (%)	0	1 (1.4%)	
Haemoperitoneum, *n* (%)	0	1 (1.4%)	
Pulmonary oedema ^@^, *n* (%)	0	1 (1.4%)	

Some patients had more than one complication; ** Placental abruption occurred in two patients. ^ Obstetric haemorrhage was defined as ≥ 500 mL estimated blood loss from the reproductive tract in the period during and after birth. ^&^ MET = medical emergency team. 21 MET calls occurred in 13 patients; ^@^ Two different patients develop pulmonary oedema—one patient preoperatively and one postoperatively; SD = standard deviation; N/A = not applicable as there were zero events in one group. ^@@^ Proportion comparisons between groups (total intraoperative and total postoperative complications) used Fisher’s exact test. *p* values are the results of comparative testing between total intraoperative complications between patients without and with severe features of preeclampsia, and total postoperative complications between patients without and with severe features of preeclampsia.

**Table 3:** Maternal complications after hospital discharge.

	No Severe Features *n* = 17	Severe Features *n* = 55	*p* Value
**Readmission to hospital, *n* (%)**	2 (2.8%)	4 (5.6%)	0.6215 ^@@^
Hypertension, *n* (%)	0	4 (5.6%)	
Infection, *n* (%)	2 (2.8%)	0	
**Presentations to ED, *n* (%)**	3 (4.2%)	12 (16.7%)	>0.9999 ^@@^
Hypertension, *n* (%)	1 (1.4%)	4 (5.6%)	
Infective symptoms, *n* (%)	0	2 (2.8%)	
Abdominal pain, *n* (%)	0	3 (4.2%)	
Vaginal bleeding/discharge, *n* (%)	0	1 (1.4%)	
Wound complications, *n* (%)	2 (2.8%)	0	
Dizziness, *n* (%)	0	1 (1.4%)	
Breast pain, *n* (%)	0	1 (1.4%)	
Midwifery review problems *, *n* (%)	4 (5.6%)	16 (22.2%)	0.7640 ^@@^
Hypertension, *n* (%)	1 (1.4%)	12 (16.7%)	
Tachycardia, *n* (%)	1 (1.4%)	0	
Wound erythema, *n* (%)	0	1 (1.4%)	
General pain, *n* (%)	0	1 (1.4%)	
Social issues, *n* (%)	1 (1.4%)	0	
Urinary tract infection, *n* (%)	0	1 (1.4%)	
Concerns about baby’s growth, *n* (%)	1 (1.4%)	1 (1.4%)	

ED = emergency department; * Problems identified at midwifery review (home visit). ^@@^ Proportion comparisons between groups for the total number (readmission to hospital, presentations to the ED and midwifery review problems) used Fisher’s exact test.

## Data Availability

Data are contained within the article.
